# Supplementary figures and images for: Arrhythmia classification for non-experts using infinite impulse response (IIR)-filter-based machine learning and deep learning models of the electrocardiogram
Source: PeerJ Comput Sci. 2024 Jan 24;10:e1774. doi: 10.7717/peerj-cs.1774 (PMC10909216; doi:10.7717/peerj-cs.1774)

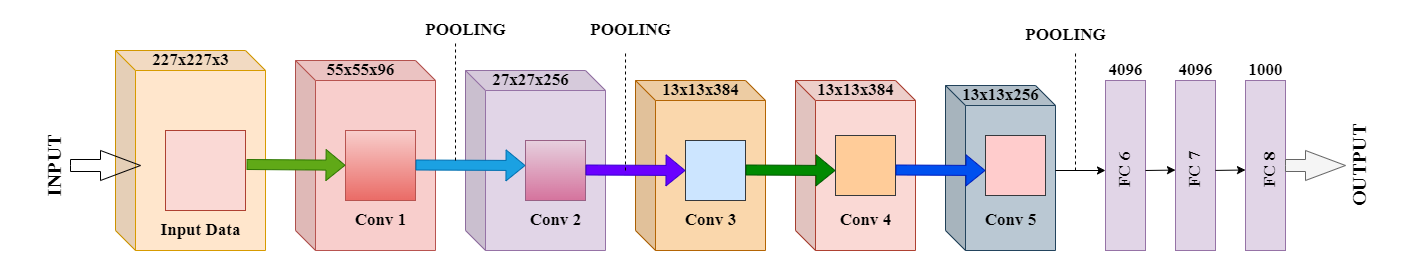

Supplement: Supplemental Information 1 [file peerj-cs-10-1774-s001.zip › Arrhythmia-Classification-main/images/2.png]

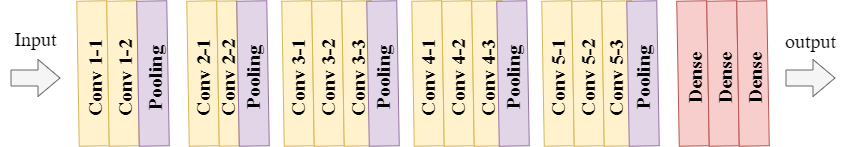

Supplement: Supplemental Information 1 [file peerj-cs-10-1774-s001.zip › Arrhythmia-Classification-main/images/3.3.png]

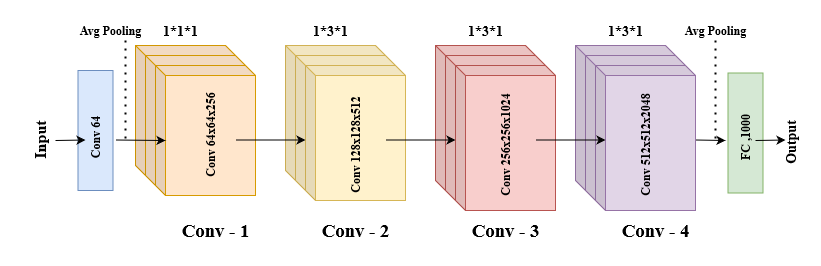

Supplement: Supplemental Information 1 [file peerj-cs-10-1774-s001.zip › Arrhythmia-Classification-main/images/4.png]

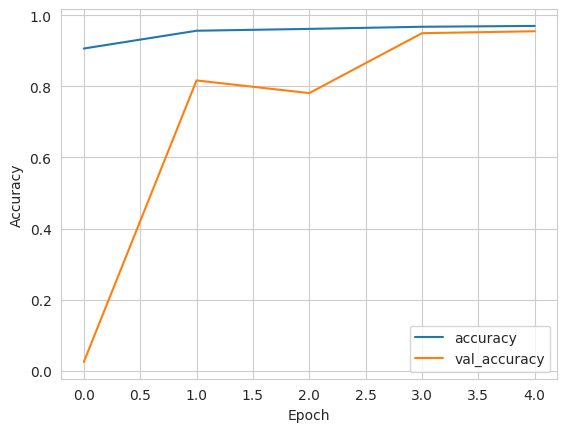

Supplement: Supplemental Information 1 [file peerj-cs-10-1774-s001.zip › Arrhythmia-Classification-main/images/ALEXNET ACCURACY.png]

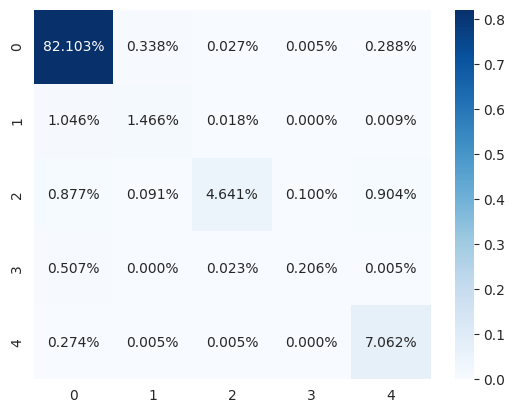

Supplement: Supplemental Information 1 [file peerj-cs-10-1774-s001.zip › Arrhythmia-Classification-main/images/ALEXNET CONFUSIONMATRIX.png]

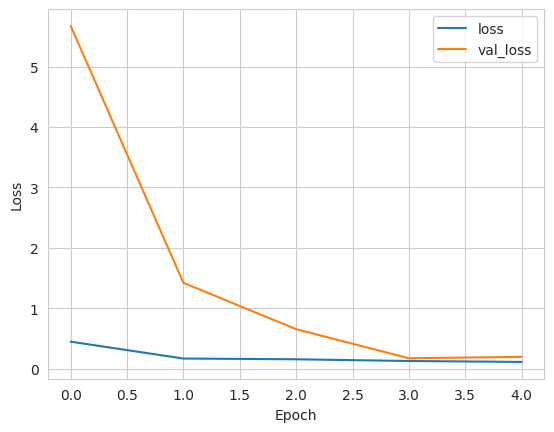

Supplement: Supplemental Information 1 [file peerj-cs-10-1774-s001.zip › Arrhythmia-Classification-main/images/ALEXNET LOSSCURVE.png]

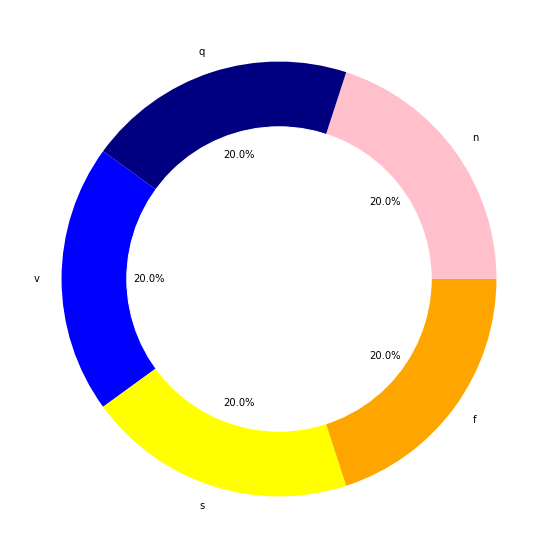

Supplement: Supplemental Information 1 [file peerj-cs-10-1774-s001.zip › Arrhythmia-Classification-main/images/BL.png]

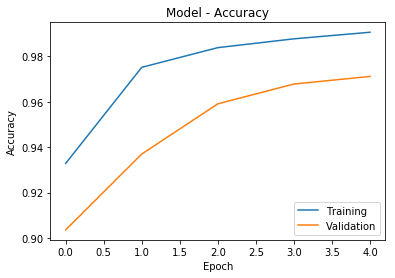

Supplement: Supplemental Information 1 [file peerj-cs-10-1774-s001.zip › Arrhythmia-Classification-main/images/DENSENET_ACCURACY.png]

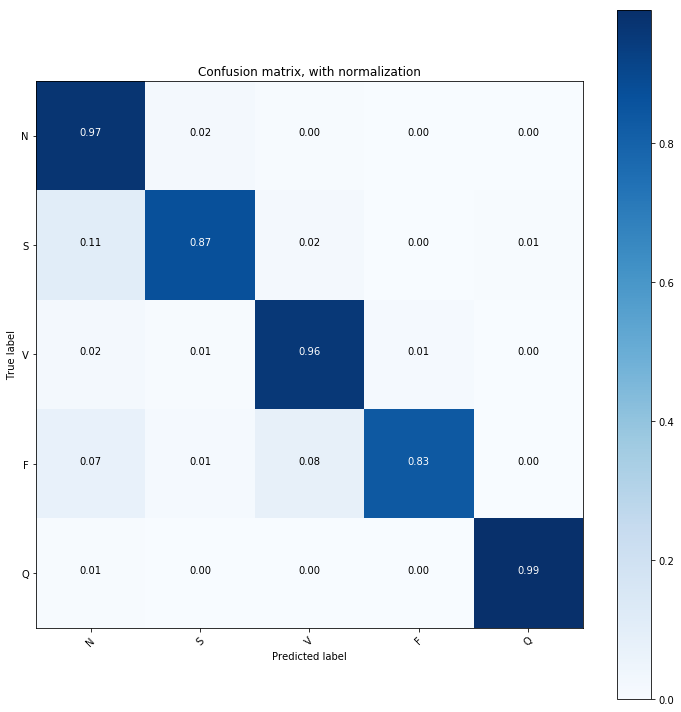

Supplement: Supplemental Information 1 [file peerj-cs-10-1774-s001.zip › Arrhythmia-Classification-main/images/DENSENET_CONFUSIONMATRIX.png]

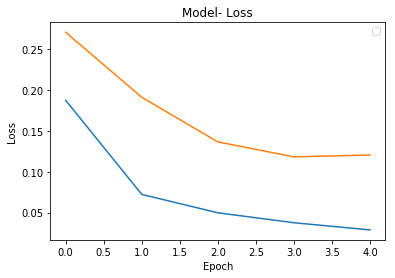

Supplement: Supplemental Information 1 [file peerj-cs-10-1774-s001.zip › Arrhythmia-Classification-main/images/DENSENET_LOSSCURVE.png]

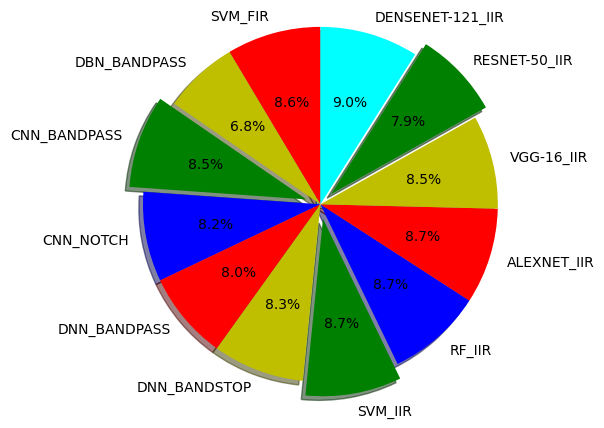

Supplement: Supplemental Information 1 [file peerj-cs-10-1774-s001.zip › Arrhythmia-Classification-main/images/FILTERTYPES.png]

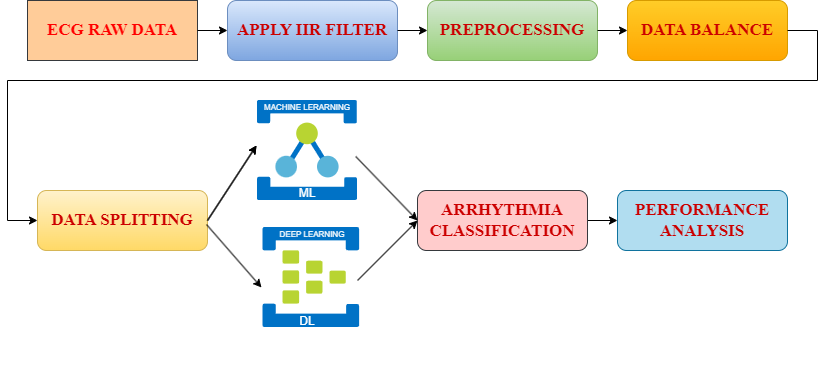

Supplement: Supplemental Information 1 [file peerj-cs-10-1774-s001.zip › Arrhythmia-Classification-main/images/MODEL3.png]

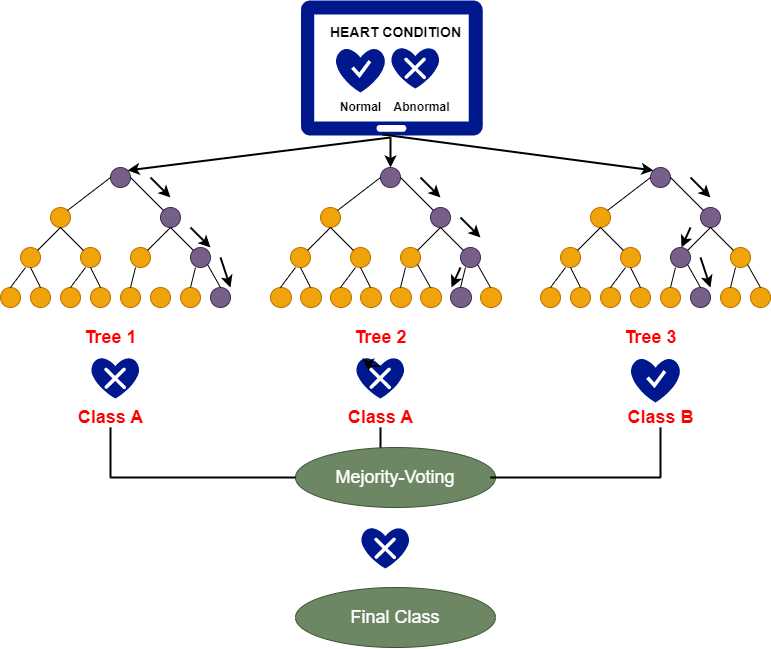

Supplement: Supplemental Information 1 [file peerj-cs-10-1774-s001.zip › Arrhythmia-Classification-main/images/RF.png]

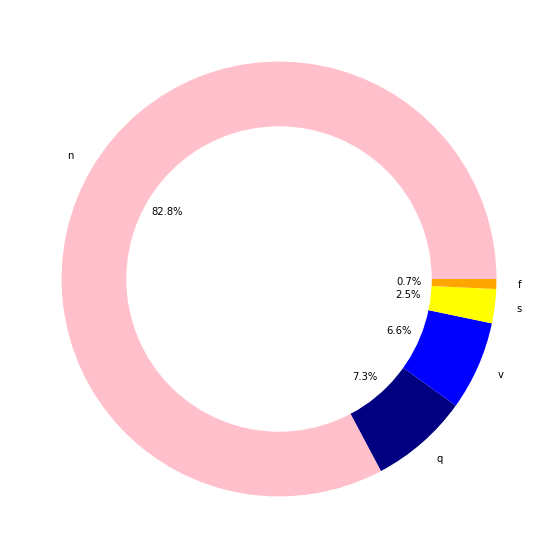

Supplement: Supplemental Information 1 [file peerj-cs-10-1774-s001.zip › Arrhythmia-Classification-main/images/UBL.png]

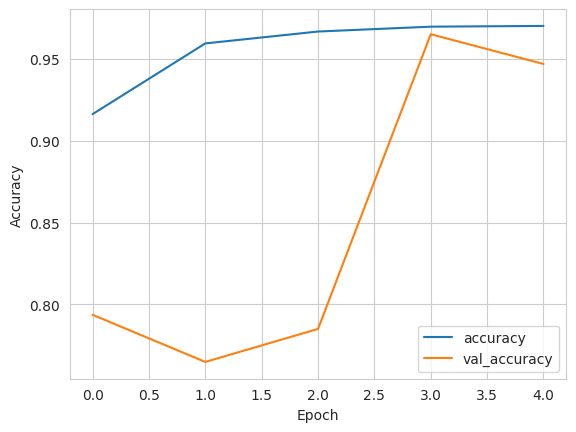

Supplement: Supplemental Information 1 [file peerj-cs-10-1774-s001.zip › Arrhythmia-Classification-main/images/VGG ACCURACY.png]

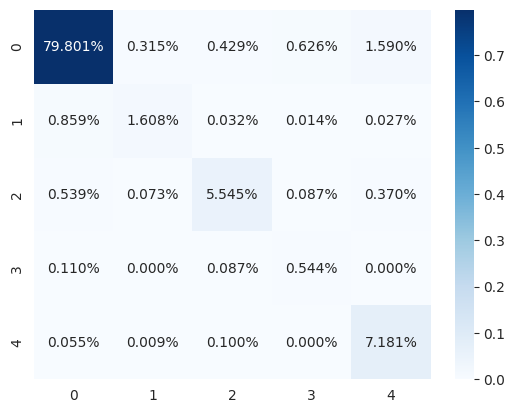

Supplement: Supplemental Information 1 [file peerj-cs-10-1774-s001.zip › Arrhythmia-Classification-main/images/VGG CONFUSIONMATRIX.png]

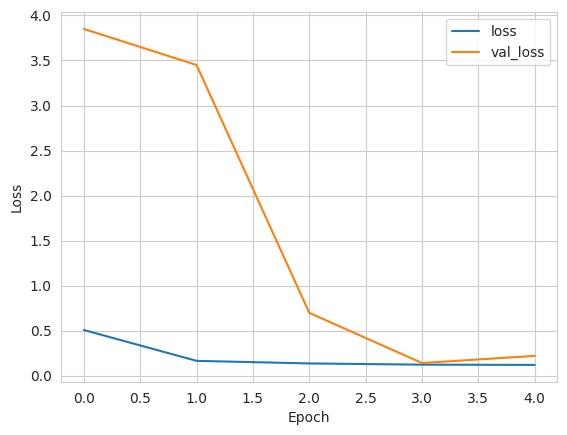

Supplement: Supplemental Information 1 [file peerj-cs-10-1774-s001.zip › Arrhythmia-Classification-main/images/VGG LOSSCURVE.png]

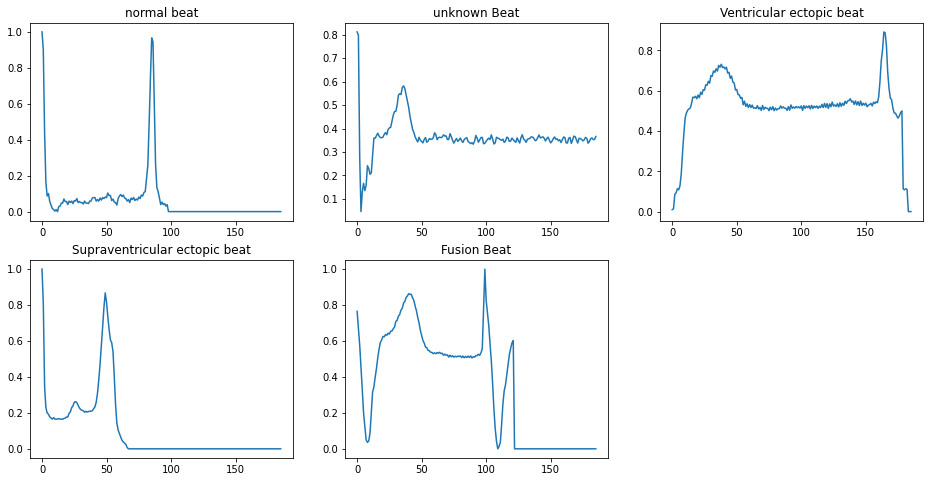

Supplement: Supplemental Information 1 [file peerj-cs-10-1774-s001.zip › Arrhythmia-Classification-main/images/classes.png]

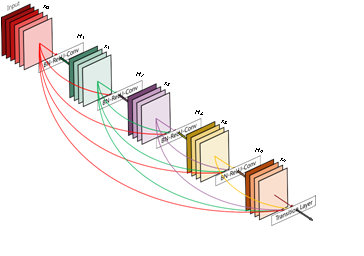

Supplement: Supplemental Information 1 [file peerj-cs-10-1774-s001.zip › Arrhythmia-Classification-main/images/densenet.png]

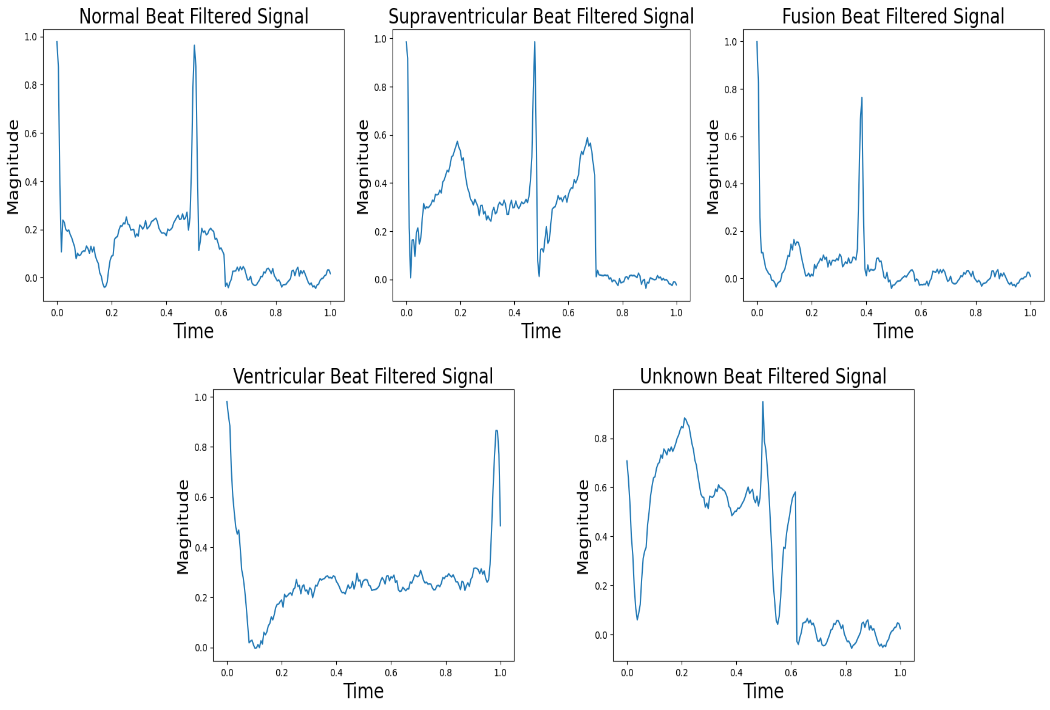

Supplement: Supplemental Information 1 [file peerj-cs-10-1774-s001.zip › Arrhythmia-Classification-main/images/fileredd.png]

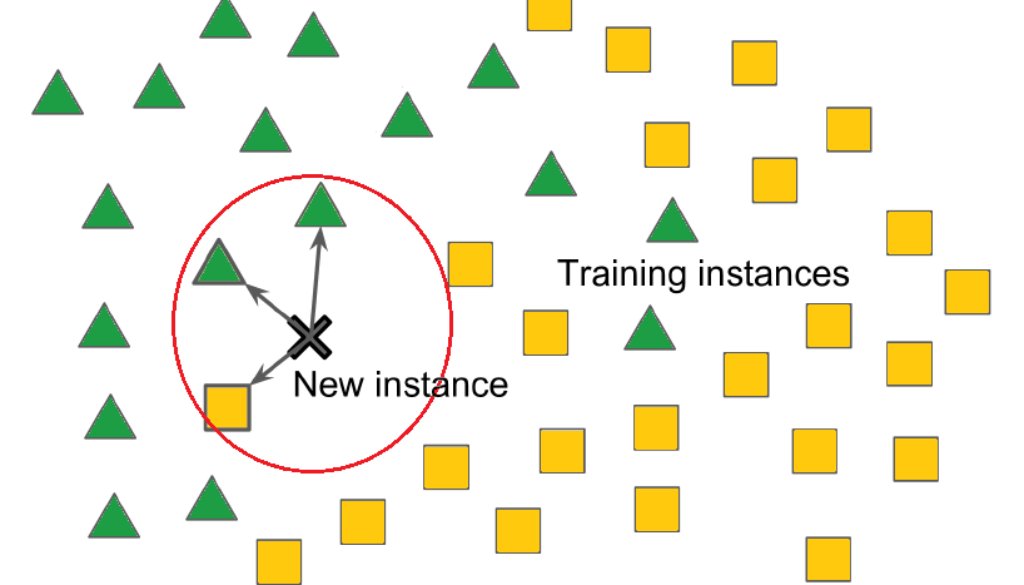

Supplement: Supplemental Information 1 [file peerj-cs-10-1774-s001.zip › Arrhythmia-Classification-main/images/knn2.jpg]

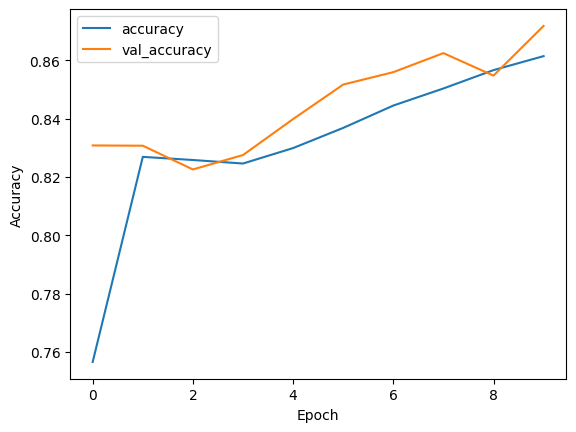

Supplement: Supplemental Information 1 [file peerj-cs-10-1774-s001.zip › Arrhythmia-Classification-main/images/resnet_accuracy.png]

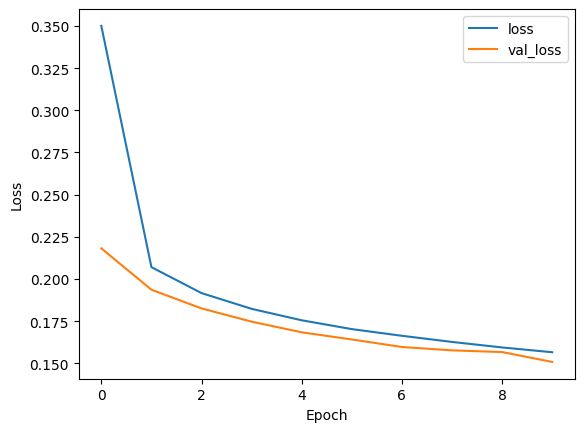

Supplement: Supplemental Information 1 [file peerj-cs-10-1774-s001.zip › Arrhythmia-Classification-main/images/resnet_losscurve.png]

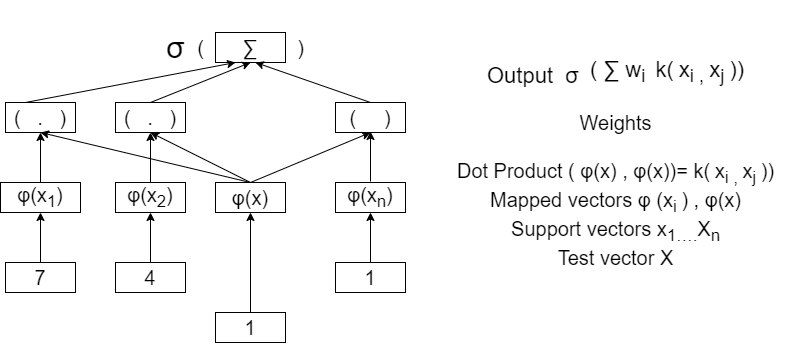

Supplement: Supplemental Information 1 [file peerj-cs-10-1774-s001.zip › Arrhythmia-Classification-main/images/svm.png]
